# Improvement of optical transmittance and electrical properties for the Si quantum dot-embedded ZnO thin film

**DOI:** 10.1186/1556-276X-8-439

**Published:** 2013-10-23

**Authors:** Kuang-Yang Kuo, Chuan-Cheng Liu, Pin-Ruei Huang, Shu-Wei Hsu, Wen-Ling Chuang, You-Jheng Chen, Po-Tsung Lee

**Affiliations:** 1 Department of Photonics and Institute of Electro-Optical Engineering, National Chiao Tung University, 1001 Ta-Hsueh Road, Hsinchu 30010, Taiwan

**Keywords:** Si quantum dot, ZnO thin film, Transport mechanism

## Abstract

A Si quantum dot (QD)-embedded ZnO thin film is successfully fabricated on a p-type Si substrate using a ZnO/Si multilayer structure. Its optical transmittance is largely improved when increasing the annealing temperature, owing to the phase transformation from amorphous to nanocrystalline Si QDs embedded in the ZnO matrix. The sample annealed at 700°C exhibits not only high optical transmittance in the long-wavelength range but also better electrical properties including low resistivity, small turn-on voltage, and high rectification ratio. By using ZnO as the QDs’ matrix, the carrier transport is dominated by the multistep tunneling mechanism, the same as in a n-ZnO/p-Si heterojunction diode, which clearly differs from that using the traditional matrix materials. Hence, the carriers transport mainly in the ZnO matrix, not through the Si QDs. The unusual transport mechanism using ZnO as matrix promises the great potential for optoelectronic devices integrating Si QDs.

## Background

Recently, Si quantum dots (QDs) embedded in traditional Si-based dielectric matrix materials like SiO_2_ and Si_3_N_4_ have been extensively studied and successfully applied to various optoelectronic devices [1-3], owing to their many unique characteristics such as highly tunable bandgap and better optical properties [4-6]. In particular, Si QD is persistently considered as a candidate for next-generation light emitters in Si photonics because of its greatly improved internal and external quantum efficiencies [7,8]. To further improve the device performance, utilization of Si-rich Si-based dielectric materials as Si QDs’ matrices has also been developed [9,10]. A suitable matrix material for Si QDs is very important for better device performance. We propose to embed Si QDs into a ZnO thin film because ZnO has many desirable features to function as Si QDs’ matrix material, e.g., wide and direct bandgap, high transparency, and highly tunable electrical properties [11]. Hence, ZnO can serve as the Si QDs’ matrix to achieve bandgap engineering, reduce the optical loss from the matrix’s absorption, and efficiently enhance the carrier transport efficiency for optoelectronic device application. The fabrication and fundamental optical properties of the Si QD-embedded ZnO thin films have been reported in our previous works [12,13]. In this study, improvement of optical transmittance and electrical properties of the Si QD-embedded ZnO thin films is investigated and discussed.

## Methods

The ZnO/Si multilayer (ML) thin films with 20 bilayers are deposited on p-type Si (100) substrates or fused quartzes at room temperature using the radio-frequency (RF) magnetron sputtering method. The sputtering powers of ZnO and Si are fixed at 75 and 110 W, and the effective thicknesses of each ZnO and Si layer are fixed at 5 and 3 nm, respectively. After deposition, the ZnO/Si ML thin films are annealed at 500°C, 600°C, 700°C, or 800°C for 30 min in N_2_ environment. For electrical measurements, 100-nm-thick Al and Ni metal layers are deposited on the top and bottom surfaces of devices as electrodes using a thermal coater. The Raman spectra are measured using a 488-nm diode-pumped solid-state laser (HORIBA LabRam HR, HORIBA, Kyoto, Japan). The X-ray diffraction (XRD) patterns are examined by a Bede-D1 X-ray diffractometer with Cu Kα radiation (Bede Scientific, Engelwood, CO, USA). The transmittance spectra are obtained using a UV–vis-NIR spectrophotometer (Hitachi U-4100, Hitachi Ltd., Chiyoda, Tokyo, Japan). The cross-sectional morphologies are observed by a JSM-6500 F field-emission scanning electron microscope (SEM; JEOL Ltd., Akishima, Tokyo, Japan). The current–voltage (*I*-*V*) curves are measured using an Agilent E5270B precision measurement mainframe (Agilent Technologies Inc., Santa Clara, CA, USA).

## Results and discussion

**Figure 1:**
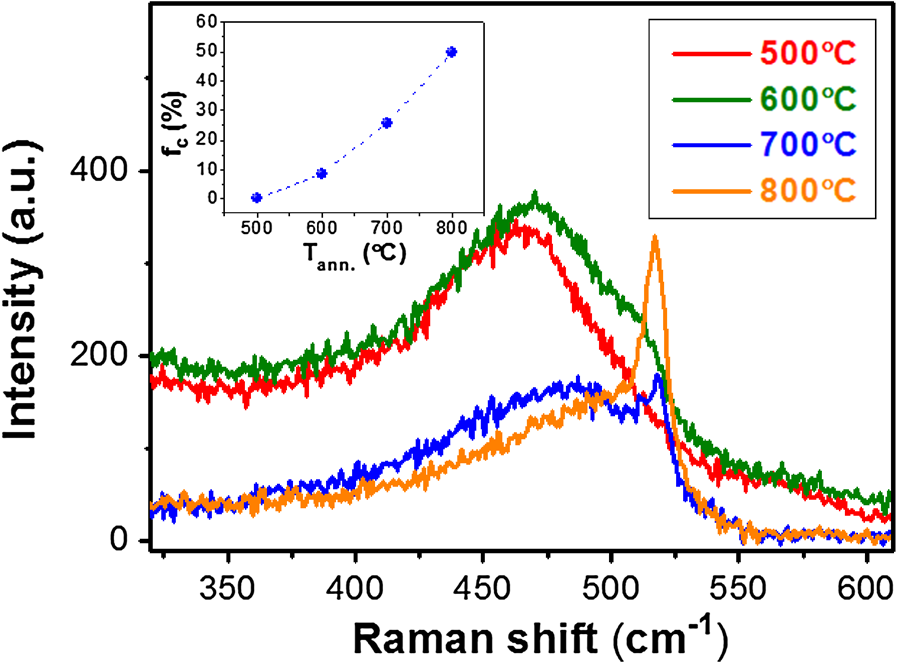
**Crystalline properties of Si QDs.** Raman spectra of the Si QD-embedded ZnO thin films under different *T*_ann_. The inset shows the corresponding crystalline volume fractions of Si (*f*_c_).

In our previous works, we demonstrated that a high Si sputtering power can assist the formation of the self-aggregated amorphous Si QDs embedded in the ZnO matrix during deposition by utilizing a ZnO/Si ML structure [12]. In order to investigate the crystalline properties of the Si QDs embedded in ZnO thin films under different annealing temperatures (*T*_ann_) for a longer annealing duration, Raman spectra are measured and shown in Figure 1. Generally, the signal of Si materials can be decomposed into three components including the peaks located at approximately 480, 500 ~ 510, and 510 ~ 520 cm^-1^, which originated from the transverse optical (TO) modes of Si-Si vibrations in the amorphous (a-Si), intermediate (i-Si), and nanocrystalline Si (nc-Si) phases [14]. The corresponding crystalline volume fractions of Si (*f*_c_) obtained from fitting the curves are shown in the inset of Figure 1[14]. The nc-Si phase is formed in the ZnO matrix and significantly increased by increasing *T*_ann_ when *T*_ann_ is higher than 600°C. This indicates that a higher *T*_ann_ can largely enhance the crystalline quality of Si QDs embedded in the ZnO matrix.

**Figure 2:**
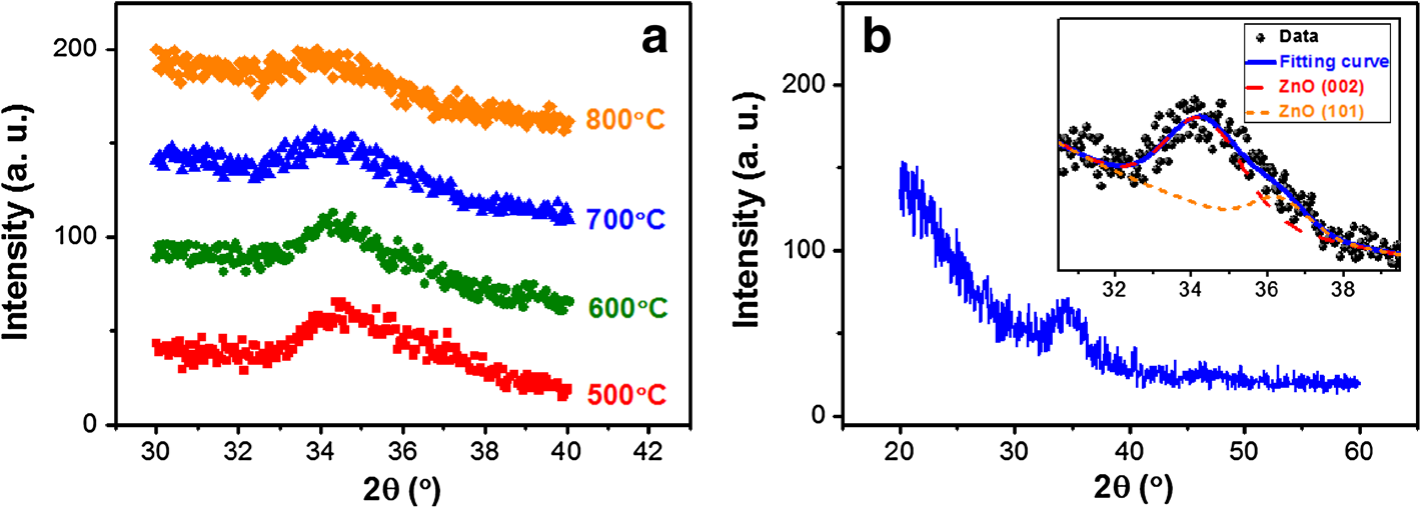
**Crystalline properties of ZnO matrix. (a)** XRD patterns fine-scanned from 30° to 40° of the Si QD-embedded ZnO thin films under different *T*_ann_. **(b)** Full XRD pattern of the Si QD-embedded ZnO thin film annealed at 700°C. The inset shows the curve fitting result for the main diffraction signal.

Since the crystalline properties of the ZnO matrix can influence its optical and electrical properties [15], the XRD patterns of the Si QD-embedded ZnO thin films annealed at different temperatures are examined and shown in Figure 2a, fine-scanned from 30° to 40°. A main diffraction signal is observed at approximately 34.5° for all the samples. As shown in Figure 2b and its inset, this signal can be decomposed into two components in Gaussian form with peaks located at about 34.3° and about 36.3°, which are contributed from (002) and (101) orientations of ZnO [16]. In Figure 2a, the crystallization intensity of the ZnO matrix is slightly reduced when increasing *T*_ann_. This may be due to the increased interior film stress resulting from the phase transformation of a- to nc-Si QDs. From the results of Raman and XRD measurements, we show that the nc-Si QDs embedded in the crystalline ZnO matrix can be achieved by a *T*_ann_ higher than 600°C.

**Figure 3:**
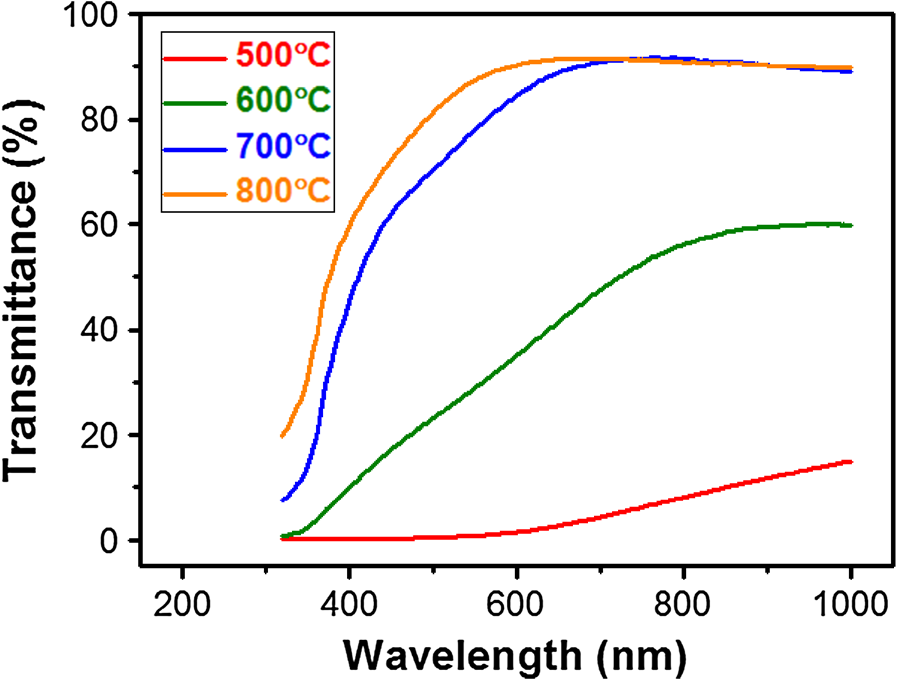
**Optical properties.** Optical transmittance spectra of the Si QD-embedded ZnO thin films under different *T*_ann_.

The optical transmittance spectra of the Si QD-embedded ZnO thin films under different *T*_ann_ are shown in Figure 3. The transmittance in the long-wavelength (long-*λ*) range (>600 nm) clearly increases when increasing *T*_ann_. Since higher *T*_ann_ can obviously enhance the crystallization of Si QDs, the improved optical transmittance in the long-*λ* range can be attributed to the decreased absorbance from a-Si QDs due to the increased *f*_c_ of Si QDs [5]. It is worthy to note that high transmittance of approximately 90% in the long-*λ* range under a *T*_ann_ higher than 700°C can be achieved and is close to those using the traditional matrix materials [5]. From the above results, it is clear that better crystallization of Si QDs in the ZnO matrix is required to decrease the absorption from a-Si QDs and thus efficiently reduce the optical loss in the long-*λ* range for better optoelectronic device performance. We find that a high-enough *T*_ann_ for the Si QD-embedded ZnO thin film is critical to significantly improve the optical properties.

**Figure 4:**
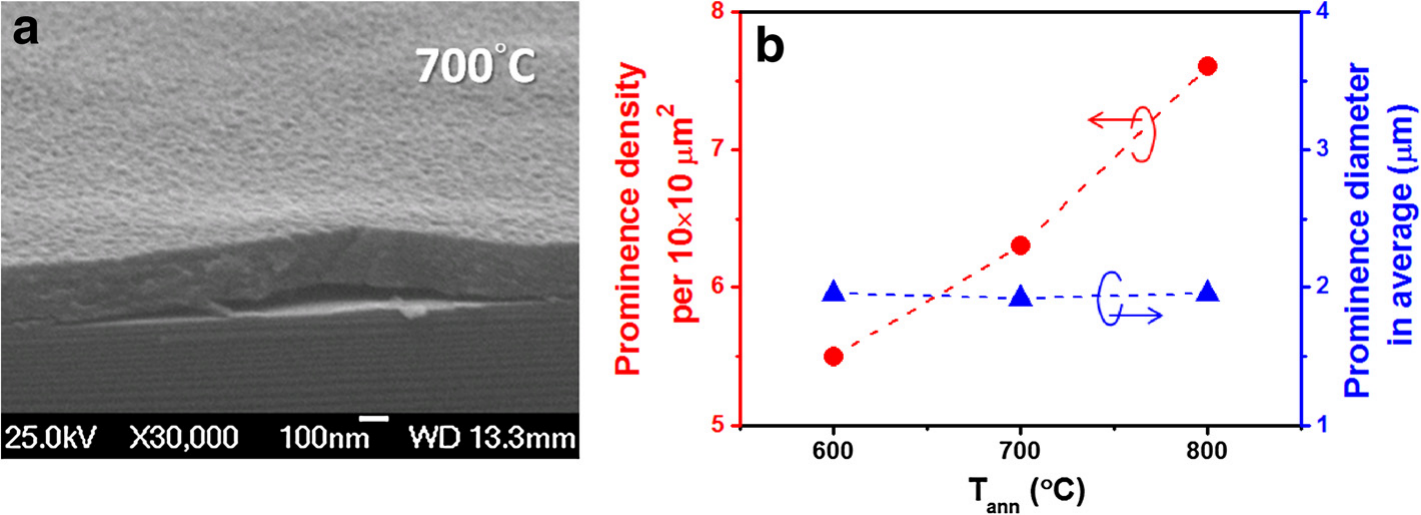
**Thin film image. (a)** Cross-sectional SEM image and **(b)** local film prominence density and diameter in average for the Si QD-embedded ZnO thin films after annealing.

The images of the Si QD-embedded ZnO thin films after annealing are examined by SEM. The local film prominences are observed when *T*_ann_ is higher than 600°C. Figure 4a shows the cross-sectional SEM image of a sample annealed at 700°C. The local film prominence density and diameter in average are estimated and shown in Figure 4b. The prominence density increases almost linearly from 5.5 to 7.6 ones per 10 × 10 μm^2^ when increasing *T*_ann_ from 600°C to 800°C with a close average diameter of about 2 μm. According to Raman spectra, the phase transformation of a- to nc-Si QDs happens when *T*_ann_ is larger than 600°C, and *f*_c_ also increases with increasing *T*_ann_ from 600°C to 800°C. This strong correlation between *f*_c_ and prominence density means that the volume variation from the phase transformation of a- to nc-Si QDs embedded in the ZnO matrix during annealing can produce an interior film stress and lead to the occurrence of local film prominences.

**Figure 5:**
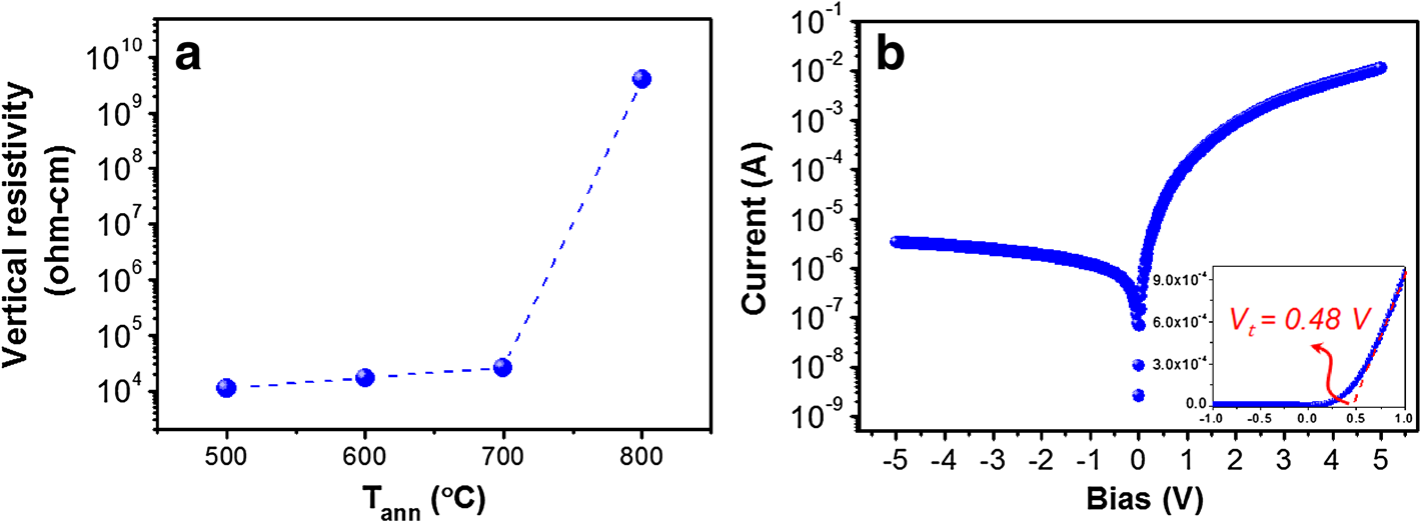
**Electrical properties. (a)** Vertical resistivity of the Si QD-embedded ZnO thin films under different *T*_ann_. **(b)** Logarithmic *I*-*V* curve of the sample annealed at 700°C. The inset shows the linear *I*-*V* curve in magnification.

To understand the electrical properties of the Si QD-embedded ZnO thin films, the vertical resistivity (*ρ*) is calculated from the slope of the *I*-*V* curve under a high forward bias region of 4 ~ 5 V. When increasing *T*_ann_, the *ρ* can be reduced by the improved crystalline quality of Si QDs but also raised by the increased film prominence density and degraded crystalline quality of the ZnO matrix. Figure 5a shows the obtained *ρ* under different *T*_ann_, which slightly increases when increasing *T*_ann_ from 500°C to 700°C but still keeps a low resistivity of approximately 10^4^ Ω cm, significantly lower than that (approximately 10^8^ Ω cm) of the intrinsic Si QDs embedded in a SiO_2_ matrix [17,18]. It is evident that using ZnO as matrix can overcome the issue of highly resistive nature of the traditional Si-based dielectric matrix materials, and 10^4^ times improvement of *ρ* is obtained. The *ρ* largely increases for the sample annealed at 800°C, which may have resulted from the excess film prominences produced during annealing since the film prominences will lead to local broken circuit regions at the interface of film/substrate and significantly increase the resistivity. Hence, we can conclude that annealing at 700°C is a more suitable annealing condition to have better crystallization of Si QDs in the crystalline ZnO matrix, low *ρ*, and high transmittance in the long-*λ* range. The logarithmic *I*-*V* curve of the sample annealed at 700°C is shown in Figure 5b, and its inset shows the corresponding linear *I*-*V* curve in magnification. It clearly exhibits not only a good rectification ratio of 3.4 × 10^3^ at ±5 V but also a low turn-on voltage (*V*_t_) of 0.48 V, which agrees with the reported results of the n-ZnO/p-Si heterojunction (HJ) diode [19,20]. Even though the Si QDs are embedded in the ZnO matrix, we show that the fabricated ZnO thin film on p-Si can still possess good p-n HJ diode behavior with large rectification ratio and low *V*_t_.

**Figure 6:**
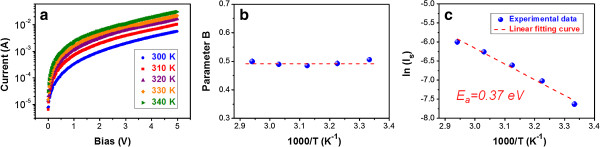
**Carrier transport mechanism. (a)** Forward *I*-*V* curves for different measurement temperatures, **(b)** the parameter *B*, and **(c)** Arrhenius plot of ln(*I*_s_) versus 1,000/T for the Si QD-embedded ZnO thin film annealed at 700°C.

To investigate the carrier transport mechanism, the temperature-dependent forward *I*-*V* curves of the sample annealed at 700°C are examined and shown in Figure 6a. The *I*-*V* curves exhibit the typical temperature dependence of a p-n junction diode. The current clearly increases as we raise the measurement temperature (*T*_meas_). In the low bias region (smaller than approximately 0.5 V), the currents can be well fitted to be proportional to about *V*^1.2^ for different *T*_meas_, which slightly deviates from the ohmic behavior. This means that the surface states and/or an inherent insulating SiO_2_ thin layer at the interface of the n-ZnO matrix/p-Si substrate has influence on the transport of carriers [21]. In the high bias region (larger than approximately 0.5 V), the forward currents can be well expressed by *I* = *I*_s_[exp(*BV*) - 1] for different *T*_meas_, where *I*_s_ is the reverse saturation current and parameter *B* is a coefficient dependent or independent on temperature decided by the dominant carrier transport mechanism [21,22]. The fitted results for parameter *B* are shown in Figure 6b, which reveal that the parameter *B* is almost invariant for different *T*_meas_. This independence of *T*_meas_ indicates that the carrier transport is dominated by the multistep tunneling mechanism, which had been reported by Zebbar et al. and Dhananjay et al. for the n-ZnO/p-Si HJ diode [21,23]. The multistep tunneling process usually occurs at the HJ region of the n-ZnO matrix and p-Si substrate, which is attributed to the recombination of electrons, tunneling from ZnO into the empty gap states in the p-Si substrate, and holes, tunneling through the HJ barrier from the p-Si substrate to the n-ZnO matrix between the empty states [21,23]. Hence, our results show that the carriers in the Si QD-embedded ZnO thin film mainly transport via the ZnO matrix but not through Si QDs with direct, resonant, or phonon-assisted tunneling mechanisms, as reported for Si QDs embedded in the traditional matrix materials [24,25]. According to the multistep tunneling mechanism, the temperature dependence of *I*_s_ is given by the relation, *I*_s_ ∝ exp(-*E*_a_/*kT*), where *E*_a_ is the activation energy, *k* is Boltzmann’s constant, and *T* is the absolute temperature [21,23]. Figure 6c shows the Arrhenius plot of ln(*I*_s_) versus 1,000/*T*. A linear relationship is clearly observed, which further confirms that the dominating carrier transport process is the multistep tunneling mechanism [19,21-23]. The *E*_a_ of around 0.37 eV obtained from the Arrhenius plot is a little larger than those of the reported n-ZnO/p-Si HJ diodes, which are usually smaller than 0.3 eV [19,21-23]. This means that the thermally activated carriers are partially contributed from the embedded Si QDs since the intrinsic Si QDs can possess *E*_a_ larger than 0.4 eV [17,26]. Thus, we can conclude that the Si QDs embedded in ZnO matrix also contribute the carriers, and those carriers will partially escape from Si QDs into the ZnO matrix and transport inside. The largely improved resistivity suggests that the carriers transporting in the ZnO matrix can have a much better transport efficiency than those tunneling through barriers in the traditional matrix materials. With the unique carrier transport mechanism and better electrical properties, we believe that the Si QD thin films will have great potential for optoelectronic device application by using ZnO as matrix material.

## Conclusions

In summary, we successfully fabricate a nc-Si QD-embedded ZnO thin film on a p-Si substrate using a ZnO/Si ML deposition structure. Our results indicate that the optical transmittance can be largely enhanced by increasing *T*_ann_ owing to the phase transformation of a- to nc-Si QDs embedded in the ZnO matrix, and up to about 90% transmittance in the long-*λ* range under a *T*_ann_ higher than 700°C is obtained. The Si QD-embedded ZnO thin film annealed at 700°C exhibits good diode behavior with a large rectification ratio of approximately 10^3^ at ±5 V and significantly lower resistivity than that using the SiO_2_ matrix material (10^4^ times improvement). From temperature-dependent *I*-*V* curves, we find that the carriers transport mainly via the ZnO matrix, not through Si QDs, which is dominated by the multistep tunneling mechanism as in the n-ZnO/p-Si HJ diode. The unique transport mechanism differing from those using the traditional Si-based dielectric matrix materials can lead to much better carrier transport efficiency and electrical properties. Hence, we show that the Si QD thin film using the ZnO matrix material is very advantageous and has potential for optoelectronics device application.

## Competing interests

The authors declare that they have no competing interests.

## Authors’ contributions

KYK and PTL carried out the experimental design and analysis and drafted the manuscript. CCL carried out the experimental fabrication and measurements. PRH, SWH, WLC, and YJC participated in the experimental fabrication. All authors read and approved the final manuscript.
